# Thyroid Hormone Receptors Predict Prognosis in *BRCA1* Associated Breast Cancer in Opposing Ways

**DOI:** 10.1371/journal.pone.0127072

**Published:** 2015-06-01

**Authors:** Sabine Heublein, Doris Mayr, Alfons Meindl, Martin Angele, Julia Gallwas, Udo Jeschke, Nina Ditsch

**Affiliations:** 1 Department of Obstetrics and Gynecology, Ludwig-Maximilians-University of Munich, Munich, Germany; 2 Department of Pathology, Ludwig-Maximilians-University of Munich, Munich, Germany; 3 Department of Obstetrics and Gynecology, Technical University of Munich, Munich, Germany; 4 Department of Surgery, Ludwig-Maximilians-University of Munich, Munich, Germany; University Claude Bernard Lyon 1, FRANCE

## Abstract

Since *BRCA1* associated breast cancers are frequently classified as hormone receptor negative or even triple negative, the application of endocrine therapies is rather limited in these patients. Like hormone receptors that bind to estrogen or progesterone, thyroid hormone receptors (TRs) are members of the nuclear hormone receptor superfamily. TRs might be interesting biomarkers - especially in the absence of classical hormone receptors. The current study aimed to investigate whether TRs may be specifically expressed in *BRCA1* associated cancer cases and whether they are of prognostic significance in these patients as compared to sporadic breast cancer cases. This study analyzed TRα and TRβ immunopositivity in *BRCA1* associated (n = 38) and sporadic breast cancer (n = 86). Further, TRs were studied in MCF7 (*BRCA1* wildtype) and HCC3153 (*BRCA1* mutated) cells. TRβ positivity rate was significantly higher in *BRCA1* associated as compared to sporadic breast cancers (p = 0.001). The latter observation remained to be significant when cases that had been matched for clinicopathological criteria were compared (p = 0.037). Regarding *BRCA1* associated breast cancer cases TRβ positivity turned out to be a positive prognostic factor for five-year (p = 0.007) and overall survival (p = 0.026) while TRα positivity predicted reduced five-year survival (p = 0.030). Activation of TRβ resulted in down-modulation of *CTNNB1* while TRα inhibition reduced cell viability in HCC3153. However, only *BRCA1* wildtype MCF7 cells were capable of rapidly degrading TRα1 in response to T3 stimulation. Significantly, this study identified TRβ to be up-regulated in *BRCA1* associated breast cancer and revealed TRs to be associated with patients’ prognosis. TRs were also found to be expressed in triple negative *BRCA1* associated breast cancer. Further studies need to be done in order to evaluate whether TRs may become interesting targets of endocrine therapeutic approaches, especially when tumors are triple-negative.

## Introduction

Breast cancers diagnosed in patients carrying a *BRCA1* germline mutation display distinct histo-pathological as well as molecular characteristics and have been observed to differ from sporadic cases also regarding chemotherapeutic sensitivity [1]. Unlike sporadic cases, *BRCA1* associated breast cancers display a higher incidence of medullary or basal-like histology and might overexpress cell cycle stimulator genes [2–4]. The fact that *BRCA1* associated breast cancers are mostly diagnosed as being negative for either classical hormone receptors (estrogen receptor (ER), progesterone receptor (PR)), for human epidermal growth factor receptor-2 (HER2) or for all of them (so called triple negative cases), is considered to be one of the most characteristic features [2,5]. As a consequence, *BRCA1* associated breast cancers require a specially tailored therapeutic regimen, since the frequent lack of hormone receptors (ER/PR) or Her2 extensively narrows the application of (anti-)endocrine therapies [5].

Like classical steroid hormone receptors (ER/PR), which are routinely used as predictive markers, thyroid hormone receptors (TRs) are members of the nuclear hormone receptor superfamily acting via transcriptional cis-regulation of target genes. However, the exact role of thyroidal effector hormones and TRs in breast cancer remains still to be elucidated. Recently, expression of TRs has been identified in up to about 79% of breast cancer cases and furthermore TRs have been shown to be associated with clinicopathological parameters such as tumor size, grade, lymph node involvement and hormone receptor status [6]. In addition, TRs have been reported to regulate a plethora of genes including those being involved in mediating cell differentiation, proliferation and apoptosis [7,8] and were found to be predictive for patient prognosis in hepatocellular carcinoma [8]. Since patients’ TR statuses can be determined easily by immunohistochemistry and as TRs were demonstrated to be accessible targets [6,9], TRs might be novel alternative biomarkers, especially for hormone receptor negative or even triple negative breast cancer patients. With a high percentage of *BRCA1* associated breast cancers being classified as triple-negative, the assessment of TR expression in those patients may turn out to be attractive in terms of alternative treatment options, applicable especially for breast cancer patients carrying a *BRCA1* germline mutation.

So far, no report exists on immunhistochemical TR reactivity in *BRCA1* associated breast cancer. Therefore, this study aimed to investigate the presence of TRs in breast cancers diagnosed in patients carrying a *BRCA1* germline mutation as compared to sporadic (i.e. without any family or personal history of breast cancer) cases. Further, TR immunostaining was tested for association with clinicopathological parameters and patient survival in breast cancer samples obtained from *BRCA1* carrier vs. sporadic cases. Breast cancer cell lines were used to determine whether TRs are active in the case of *BRCA1* deficiency.

## Patients and Methods

### Patients and Specimen Characteristics

Breast cancer patients diagnosed with sporadic (n = 86) or *BRCA1* associated breast cancer (n = 38) were included in this study. The majority of cases were diagnosed with a tumor of non-specific type (NST, n = 96, 77.4%), high grade (G3, n = 77, 62.6%) or staged higher than pT1 (n = 78, 62.9%). A significant number of patients also presented with either lymph node (n = 66, 57.9%) or distant metastasis (n = 70, 63.1%).

Formalin fixed paraffin embedded (FFPE) breast cancer tissue was collected from patients who had undergone surgery due to a malignant tumor of the breast, either without positive family history of breast cancer (sporadic cancer cases, n = 86) or with the diagnosis of carrying a *BRCA1* germline mutation (n = 38). Breast cancer tissue was gained at surgery and underwent routine histopathological processing and examination.

### Study Design

Patients were recruited at the Department of Gynecology and Obstetrics at the Ludwig-Maximilians-University of Munich, Germany between 1987 and 2009. Women only diagnosed for benign tumors of the breast or for *in situ* carcinoma were excluded from the study. Clinical as well as follow-up data were retrieved retrospectively from patients’ charts, from the Munich Cancer Registry or by direct contact. Overall mean survival of the cohort was 7.3 years (95% CI: 6.2–8.3 years) and mean follow up time was 6.6 years (95% CI: 5.7–7.5 years). The outcome assessed was patient five-year and overall survival. Mean age (± STDV) of the cohort was 50.0 ± 13.3 years (*BRCA1* associated cases: 41.9 ± 10.8 years; sporadic breast cancer: 53.7 ± 12.8 years).

### Ethical Approval

All patient data were fully anonymized and the study was performed according to the standards set in the declaration of Helsinki 1975. All tumor tissue used was left-over material that had initially been collected for histo-pathological diagnostics. All diagnostic procedures had already been fully completed when samples were retrieved for the study. The current study was approved by the Ethics Committee of the Ludwig-Maximilians-University, Munich, Germany (approval number 048-08). Authors were blinded from the clinical information during experimental analysis.

### Assay Methods

#### Mutation Screening

Mutation screening was performed in a standardized manner at a German center for *BRCA1* mutation testing (Technical University of Munich, Munich, Germany) as described by Fischer et al. [10]. In brief, PCR products comprising all coding exons of the *BRCA1* gene were analyzed by high performance liquid chromatography (dHPLC) followed by sequencing of conspicuous amplicons or by direct sequencing of all *BRCA1* amplicons. The NCBI (National Center for Biotechnology Information) cDNA sequence U14680.1 (*BRCA1*) served as a reference. Multiplex ligation-dependent probe amplification (MLPA) was used to screen for deletions or duplications in *BRCA1* in case of negative sequencing results. So called variants of unknown significance (VUS) characterized as Class III were not considered as mutations.

#### Cell Culture

MCF7 (*BRCA1* ^wt^) and HCC3153 (*BRCA1* ^943ins10^) breast cancer cell lines were bought from the European Collection of Cell Cultures (MCF7) or were gently provided by Adi F. Gazdar (Hamon Center for Therapeutic Oncology Research and Department of Pathology, University of Texas Southwestern Medical Center, Dallas, TX) (HCC3153). While MCF7 carry a wildtype *BRCA1* allele, HCC3153 show a homozygous insertion in exon eleven of *BRCA1* (*BRCA1* ^943ins10^) leading to a premature stop codon and thus encoding a truncated BRCA1 protein [11]. Cells were cultured in DMEM (Biochrom, Berlin, Germany) containing stable glutamine and supplemented with 10% fetal bovine serum (FBS) without antibiotics/antimycotics. Mycoplasma testing was performed routinely.

Seeding densities were as follows: 24 well plate or 4-perm Chamberslide—7.5 x 10^4^ cells per well, 96 well plate—3.0 x 10^3^ cells per well (stimulation assays) and 6.0 x 10^3^ cells per well (siRNA knockdown), Quadriperm—7.5 x 10^5^ cells per well.

#### Immunostaining

**Fig 1:**
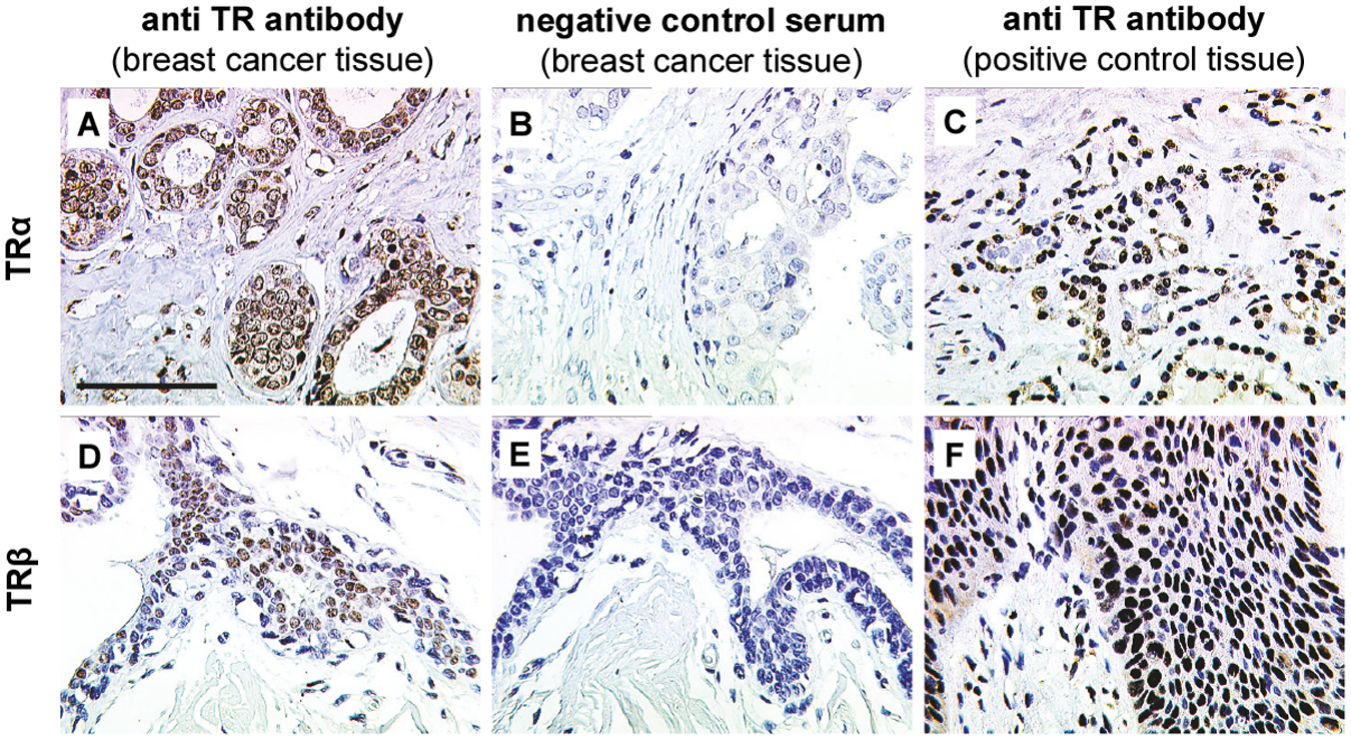
TRα and TRβ immunostaining. Positive TRα (A) and TRβ (D) staining was detected in breast cancer tissue. Negative (TRα (B) and TRβ (E)) and positive controls (TRα (C) and TRβ (F)) were performed to validate staining specificity. Thyroid gland and vaginal epithelium served as tissue positive controls for TRα (C) and TRβ (F), respectively. Scale bar represents 100 μm and applies to A-F. Representative photomicrographs are shown.

Immunohistochemistry of TRα and TRβ on FFPE sections has been extensively described by our group [6,12]. In brief, rabbit polyclonal antibodies detecting TRα (Abcam, Cambridge, UK); Zytomed, Berlin, Germany) or TRβ (Zytomed)) were stained by employing commercially available kits (Vectastain Elite rabbit-IgG-Kit (VectorLabs, Burlingame, CA); ZytoChem Plus HRP Polymer System (Zytomed). Appropriate positive (struma and vaginal tissue [6]) and negative controls were included in each experiment (Fig 1). Tissue sections treated with pre-immune IgGs (supersensitive rabbit negative control, BioGenex, Fremont, CA) instead of the primary antibody served as negative controls. Immunoreactivity was quantified by applying a well-established semiquantitaive scoring system (IR-score; also known as Remmele’s score) by two independent observers by consensus. This scoring method has already been used in numerous studies [6,13–15] and quantifies immunoreactivity by multiplication of optical staining intensity (graded as 0: no, 1: weak, 2: moderate and 3: strong staining) and the percentage of positive stained cells (0: no staining, 1: ≤ 10% of the cells, 2: 11–50% of the cells, 3: 51–80% of the cells and 4: ≥ 81% of the cells). According to previously published data tissue samples that had been assigned an IRS higher than 2 were scored as positive [16,17].

Double immunofluorescence of FFPE tissue sections was done as explained in [18] using TRα2 (Abcam) and TRβ (Zytomed. Berlin, Germany) as primary antibodies.

MCF7 (*BRCA1* ^wt^) and HCC3153 (*BRCA1* ^943ins10^) were grown on glass slides and fixed in 3.7% neutral buffered formaline (15 min). Following PBS washes and blocking in PBS 0.1% TritonX for one hour cells were stained by immunocytochemistry as described in [18] using TRα (diluted 1:200 in PBS) and TRβ (diluted 1:400 in PBS) antibodies.

For immunofluorescence cells were treated with T3 (10^-7^ M) for two hours under serum free conditions, processed as depicted above and stained for rabbit-anti-TRα1 (Abcam) or co-stained for rabbit-anti-TRα1 and for mouse-anti-ubiquitin (Enzo Life Sciences, Farmingdale, NY). Primary antibodies were diluted 1:100 and incubated overnight at 4°C. Secondary antibodies (goat-anti-rabbit-Cy2, goat-anti-mouse-Cy3; Jackson Immunolabs, West Grove, PA) were diluted 1:300 and incubated for 30 min at room temperature. Finally, samples were covered in Vectashield (VectorLabs) mounting medium containing DAPI and were imaged on a Leica confocal microscope (Leica TCS SP5 II, Leica, Wetzlar, Germany).

#### Western Blotting

Cells were seeded in 24 well plates and medium was changed to serum free DMEM two hours after plating. Cells were transfected using *THRA-*specific siRNA (si2, si3; both from Qiagen, Hilden, Germany) or scrambled control RNA (AllStars negative control, Qiagen) for 24 hours as per manufacturer’s protocol using HiPerFect (HiP) transfection reagent (Qiagen) [18] straight after changing to serum free DMEM or were stimulated with T3 (10^-7^ M for two hours) the next day. siRNAs targeting *THRA* (si2, si3, both from Qiagen, Hilden, Germany) were used. A scrambled siRNA (AllStars negative control, Qiagen) and samples treated with the transfection reagent only served as controls.

Protein samples were quantified, processed and blotted as explained elsewhere [18]. Antibodies detecting TRα1 and Histone H2B were from Abcam (Cambridge, UK) and diluted 1:500 in 2% marvel TBST. Histone H2B was used as a loading control.

#### Quantitative Real-time PCR

Total mRNA was isolated employing the NucleoSpin RNA II kit (Machery-Nagel, Düren, Germany). Having adjusted RNA concentrations cDNA synthesis was carried out as described elsewhere [19]. Gene expression of *THRA*, *THRB* and *CTNNB1* was quantified by TaqMan real-time PCR and *ACTB* was used as a housekeeping gene. PCR conditions were: 20 s at 95°C and 40 cycles of 3 s (95°C) plus 30 s (60°C) employing the following primers (all from Applied Biosystems, Carlsbad, CA): *THRA* (Hs00268470_m1), *THRB* (Hs00230861_m1), *CTNNB1* (Hs00170025_m1), *ACTB* (Hs99999903_m1). Differences in gene expression were calculated using the Rest2009 software [20] and graphs were built afterwards from Rest2009 outputs.

#### BrdU and WST-1 measurements

Cells were seeded in 96 well plates and medium was changed to DMEM w/o FBS two hours after plating. Cells were treated with 2-(2-(-(4-Nitrophenyl)-4-piperidinylidene)-acetyl-N-(3-(trifluoromethyl)phenyl)-1-hydrazine Carboxamide (1–850, Merck, Darmstadt, Germany) at concentrations of 10^-5^ M and 10^-4^ M. Controls treated with equal amounts of carrier solution (DMSO) served as controls. Cells were stimulated for six days before proliferation and viability were quantified by BrdU and WST-1 (both Roche, Penzberg, Germany), respectively. SiRNA transfections were performed as described before [18]. Cells were transfected in serum-free DMEM using siRNAs targeting *THRA* (si2, si3; both from Qiagen) while samples just treated with a scrambled siRNA (AllStars negative control, Qiagen) were included in each experiment. Cell viability was quantified by WST-1 six days after transfection.

### Statistical Analysis Methods

This study has been carried out according to the REMARK (Reporting Recommendations for Tumor Marker Prognostic Studies) criteria [21].

The IBM statistic package SPSS (version 22) was used to test data for statistical significance. Fisher’s exact test and the Mann-Whitney test were used. Survival times were compared by Kaplan-Meier graphics and differences in patient overall survival times were tested for significance by using the chi-square statistics of the log rank test. Cell culture experiments were repeated three times achieving equal results and data were assumed to be statistically different in case of p < 0.05.

Statistical analyses were done in the whole sample as well as in a group of 56 patients (n (sporadic) = 28, n (*BRCA1* associated) = 28) that had been matched (p = 1.000) according to tumor size, lymph node status, presence of metastasis and tumor grade.

## Results

### Study Cohort

Most cases investigated were diagnosed with invasive breast cancer of non-specific type (NST, n = 96, 77.4%) or of low differentiation (G3, n = 77, 62.6%). Patients carrying a *BRCA1* mutation (n = 38) were diagnosed with high grade (G3, p = 0.025), pT1 (p = 0.008) and pN0 (p = 0.040) staged breast cancer significantly more often than sporadic cancer cases (n = 86). In addition, *BRCA1* mutated and sporadic breast cancers differed regarding patient age (p < 0.001). With respect to hormone receptor expression, *BRCA1* mutant carcinomas were significantly more frequently found to be ER negative (p = 0.001), PR negative (p = 0.001) or even triple negative (p = 0.002). Interestingly, overall survival rate of patients carrying a *BRCA1* germline mutation was significantly higher than in sporadic breast cancer cases (p < 0.001). Patient characteristics and absolute numbers are listed in Table 1.

**Table 1:** Patient characteristics (whole sample).

	breast cancer type			BRCA1					sporadic breast cancer						
	BRCA1	sporadic		TRα			TRβ			TRα			TRβ		
			p	negative	positive	p	negative	positive	p	negative	positive	p	negative	positive	p
**Histology**															
non NST	8	20	ns	4	4	ns	3	5	ns	9	11	ns	15	5	ns
NST	30	66		17	13		15	15		27	39		52	14	
**Grading**															
G1, G2	8	38	0.025	2	6	ns	5	3	ns	12	26	ns	28	10	ns
G3	29	48		18	11		12	17		24	24		39	9	
**pT**															
pT1	21	25	0.008	12	9	ns	9	12	ns	14	11	ns	18	7	ns
pT2-4	17	61		9	8		9	8		22	39		49	12	
**pN**															
pN0	20	28	0.040	13	7	ns	7	13	ns	13	15	ns	19	9	ns
pN1-3	15	51		6	9		10	5		20	31		42	9	
**pM**															
pM0	18	23	ns	11	7	ns	7	11	ns	14	9	0.011	16	7	ns
pM1	18	52		8	10		11	7		15	37		41	11	
**ER**															
negative	27	27	0.001	14	13	ns	15	12	ns	12	15	ns	21	6	ns
positive	11	44		7	4		3	8		21	23		33	11	
**PR**															
negative	27	25	0.001	14	13	ns	14	13	ns	13	12	ns	21	4	ns
positive	11	46		7	4		4	7		20	26		33	13	
**Her2**															
negative	22	29	ns	10	12	ns	10	12	ns	14	15	ns	21	8	ns
positive	6	20		4	2		3	3		12	8		17	3	
**Triple negative**															
no	17	57	0.002	10	7	ns	7	10	ns	29	28	ns	43	14	ns
yes	12	7		5	7		7	5		3	4		6	1	
**Patient age**															
≤ 42 y	22	19	< 0.001	12	10	ns	8	14	ns	6	13	ns	14	5	ns
> 42 y	16	66		9	7		10	6		30	36		52	14	

To compare further *BRCA1* associated vs. sporadic cases, a second study panel of 56 patients that had been matched (p = 1.000) according to tumor size, lymph node status, presence of metastasis and tumor grade, was selected from the whole study cohort (Table 2). This matched group did not significantly differ regarding histologic subtype, ER, PR or Her2. However, patient age remained to be different in matched groups (p = 0.011).

**Table 2:** Patient characteristics (matched groups).

	BRCA1						sporadic breast cancer					
	TRα			TRβ			TRα			TRβ		
	negative	positive	p	negative	positive	p	negative	positive	p	negative	positive	p
**Histology**												
non NST	2	3	ns	3	2	ns	5	1	ns	6	0	ns
NST	12	11		13	10		11	11		18	4	
**Grading**												
G1, G2	2	5	ns	5	2	ns	3	4	ns	6	1	ns
G3	12	9		11	10		13	8		18	3	
**pT**												
pT1	7	6	ns	7	6	ns	9	4	ns	11	2	ns
pT2-4	7	8		9	6		7	8		13	2	
**pN**												
pN0	10	5	ns	6	9	ns	8	7	ns	12	3	ns
pN1-3	4	9		10	3		8	5		12	1	
**pM**												
pM0	7	4	ns	5	6	ns	8	3	ns	10	1	ns
pM1	7	10		11	6		8	9		14	3	
**ER**												
negative	9	10	ns	13	6	ns	9	8	ns	14	3	ns
positive	5	4		3	6		7	4		10	1	
**PR**												
negative	10	10	ns	12	8	ns	10	6	ns	14	2	ns
positive	4	4		4	4		6	6		10	2	
**Her2**												
negative	8	9	ns	9	8	ns	7	6	ns	11	2	ns
positive	2	2		3	1		7	2		9	0	
**Triple negative**												
no	7	7	ns	7	7	ns	12	6	ns	16	2	ns
yes	4	4		6	2		3	3		5	1	
**Patient age**												
≤ 42 y	8	7	ns	7	8	ns	3	2	ns	4	1	ns
> 42 y	6	7		9	4		13	10		20	3	

### TRs are Frequently Expressed in *BRCA1* Associated Breast Cancer

TRs were found to be expressed in breast cancer tissue through a nuclear staining signal (Fig 1A and 1D). Specificity of TR staining was controlled by incubating breast cancer tissue sections with species-matched control serum instead of the primary antibody. No staining signal was visible in these control sections (Fig 1B and 1E). Tissue sections of thyroid gland struma (Fig 1C) and vaginal epithelium (Fig 1F) constantly presented a high TR immunoreactivity and were thus chosen as positive controls.

**Fig 2:**
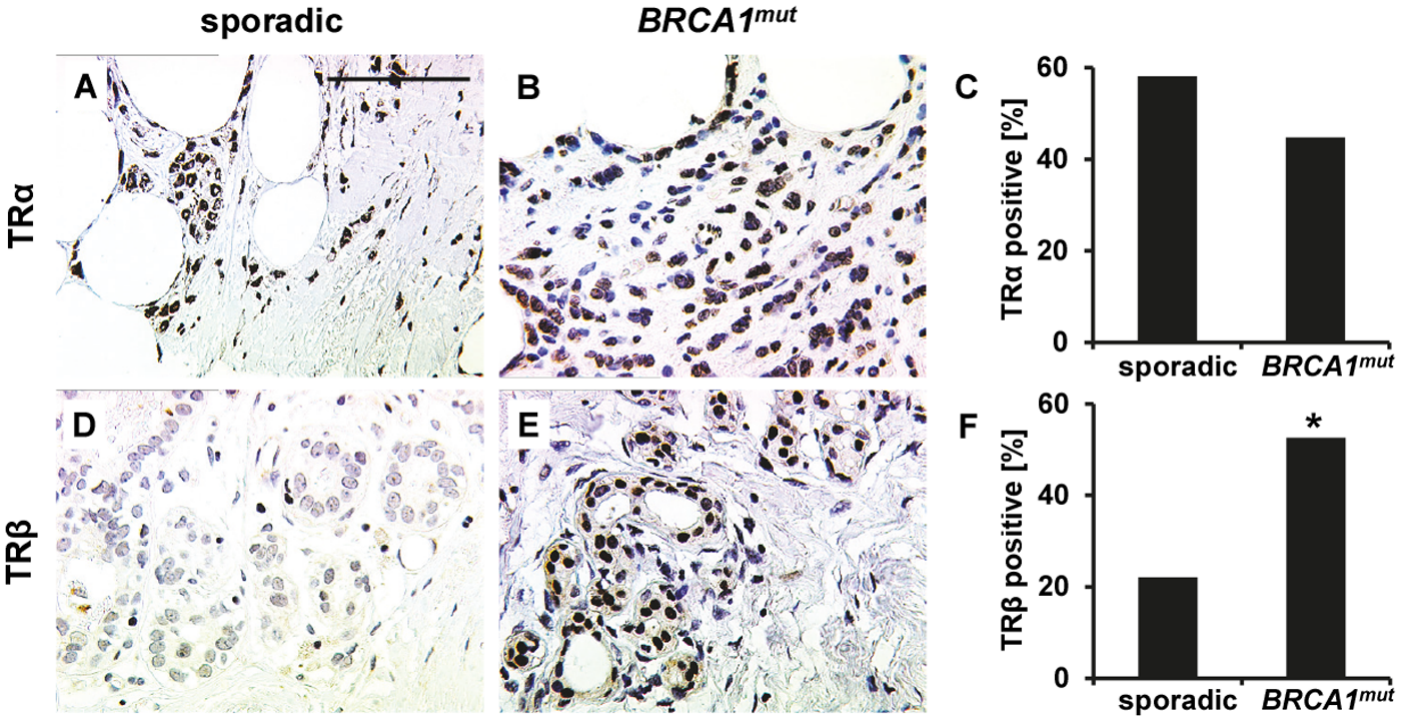
TRα and TRβ staining in breast cancer tissue. Representative photomicrographs of TRα and TRβ immunohistochemistry staining in breast cancer tissue are shown. TRα was found to be abundantly expressed though there was no significant difference regarding the number of TRα positive cases when sporadic (A) vs. *BRCA1* (B) mutated cancers were compared (C). Representative images of a TRβ staining scored as negative (D) and a TRβ staining scored as positive (E) in spontaneous (D) and *BRCA1* mutated (E) cancer tissue are presented, respectively. TRβ was found to be expressed more frequently in *BRCA1* mutated (E) cases as compared to spontaneous breast cancers (D). The fraction of TRβ positive cases in each group is illustrated in F. Significant changes are indicated by stars (*) and scale bar (applies to A, B, D and E) represents 100 μm.

**Fig 3:**
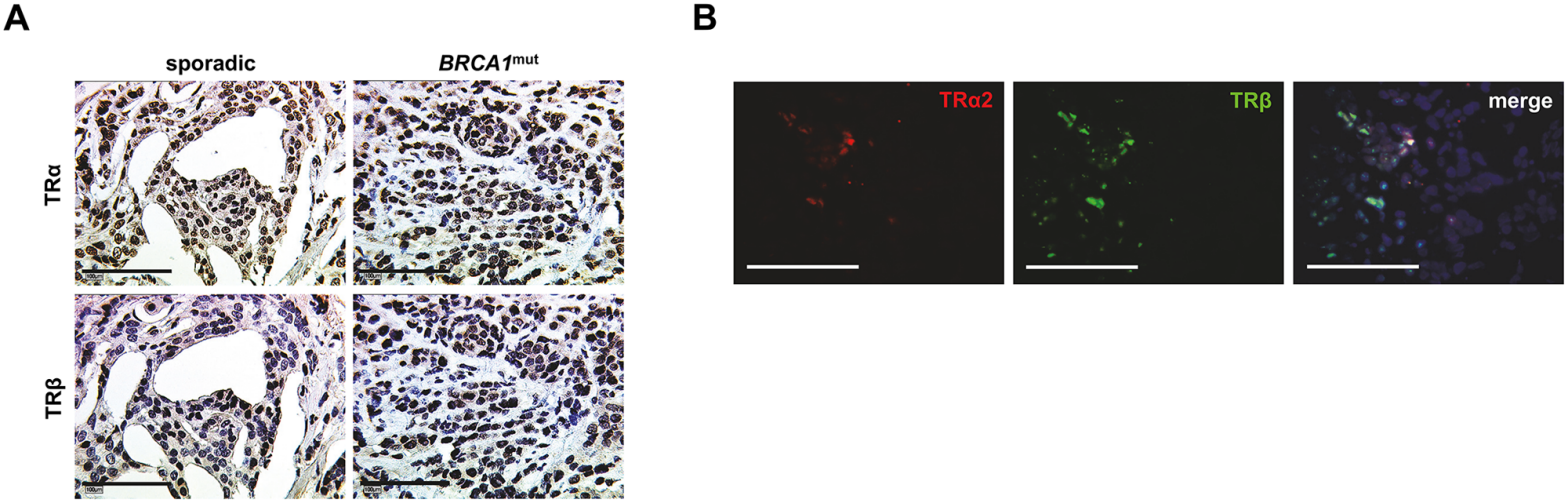
Co-localisation of TRα and TRβ. Co-localisation of TRα and TRβ was observed in some tumor cells as shown by staining serial slides (A) or by co-staining of TRs (TRα2: red signal, TRβ: green signal, DNA: blue signal) by double-immunoflourescence (B). Scale bars represent 100 μm and representative photomicrographs are shown.

Regarding sporadic cancer cases (Fig 2A and 2D) 57 out of 86 (66.3%) stained positive for at least one of the two TRs (i.e. T0052α (50/86) and/or TRβ (19/86)), 12 out of 86 (14.0%) were scored as double positive (i.e. expressing TRα and TRβ at the same time) and 33.7% (29/86) were double negative. The majority of *BRCA1* mutated tumors (30/38; 78.9%) were scored as positive for TRα and/or TRβ while only eight (8/38; 21.1%) cases expressed neither TRα nor TRβ. 17/38 (44.7%) cases stained positive for TRα (Table 3, Fig 2B and 2C), and 20/38 (52.6%) stained positive for TRβ (Table 3, Fig 2E and 2F). Finally, seven out of the 38 (18.4%) patients included were diagnosed a double positive tumor (i.e. a tumor expressing both TRα and TRβ at the same time). Regarding TRα positivity, there was no significant difference comparing sporadic (50/86; 58.1%) vs. *BRCA1* associated cases (17/38; 44.7%; Fig 2A–2C). As compared to *BRCA1* mutated cases (20/38; 52.6%) significantly fewer sporadic cancers (19/86; 22.1%) expressed TRβ (p = 0.001, Fig 2D–2F). With respect to TRβ this effect remained to be significant when cancers of high grade (p < 0.001) or of negative lymph node status (p = 0.039) were compared. Furthermore, also in the matched group, TRβ positivity was associated with the presence of mutant *BRCA1* (p = 0.037). Again, we did not observe a significant difference in receptor expression ratios in the case of TRα comparing sporadic (12/28; 42.9%) vs. *BRCA1* (14/28; 50.0%) associated cases. TRα and TRβ were not correlated among each other, neither in sporadic nor in *BRCA1* associated cases. However co-expression of both receptors as determined by staining serial sections (Fig 3A) or by double-immunofluorescence (Fig 3B) was observed in some cells. Finally, in *BRCA1* mutated cases there was no relation of TR positivity and circulating hormone levels (Table 4).

**Table 3:** Overall TR immunoreactivity.

			n	%
**total**		BRCA1	38	30.6
		sporadic	86	69.4
**BRCA1**	TRα	negative	21	55.3
		positive	17	44.7
	TRβ	negative	18	47.4
		positive	20	52.6
**sporadic breast cancer**	TRα	negative	36	41.9
		positive	50	58.1
	TRβ	negative	67	77.9
		positive	19	22.1

**Table 4:** TSH, fT3 and fT4 serum levels.

				*BRCA1*			sporαdic			*BRCA1*			sporαdic		
	*BRCA1*	sporadic	p	TRα neg.	TRα pos.	p	TRα neg.	TRα pos.	p	TRβ neg.	TRβ pos.	p	TRβ neg.	TRβ pos.	p
**TSH**															
ref. range	10	20		5	5		12	8		4	6		17	3	
< ref. range	2	4	ns	1	1	ns	3	1	ns	2	0	ns	2	2	ns
> ref. range	1	2	ns	0	1	ns	1	1	ns	0	1	ns	0	2	0.043
**fT3**															
ref. range	6	10		3	3		6	4		4	2		7	3	
< ref. range	0	0	na	0	0	na	0	0	na	0	0	na	0	0	na
> ref. range	0	0	na	0	0	na	0	0	na	0	0	na	0	0	na
**fT4**															
ref. range	7	11		3	4		8	3		4	3		8	3	
< ref. range	0	0	na	0	0	na	0	0	na	0	0	na	0	0	na
> ref. range	0	2	ns	0	0	na	1	1	ns	0	0	na	1	1	ns

TSH and thyroid hormone levels in breast cancer patients as quantified at time of first diagnosis are shown. Since clinical data were retrieved retrospectively, data regarding thyroid function were not available in all the cases. ref = reference, ns = not significant, na = not applicable

Both, ER and PR, were expressed in about one-third (ER: 11/38, PR: 11/38) of *BRCA1* mutant breast cancer. Information on Her2 was only available in 28 *BRCA1* mutated cases, and six of these 28 patients (6/28, 21.4%) were scored as Her2 positive. Sufficient information to conclude on potential presence of triple negativity was available in 29 of 38 *BRCA1* associated cancers. Twelve out of these 29 cases were finally classified as triple negative (12/29, 41.4%) while the remaining 17 cases were scored positive for at least one of the three hormone receptors (ER, PR, Her2). Interestingly, nine of these twelve (9/12, 75.0%) triple negative *BRCA1* associated breast cancer cases were found to express at least one of the two TRs. TRα was detected in seven out of twelve (7/12, 58.3%) and TRβ was detected in five of twelve (5/12, 41.7%) triple negative breast cancer cases, respectively. Three triple negative cases (3/12, 25.0%) stained positive for both TRs at the same time while another three cases (3/12, 25.0%) expressed neither TRα nor TRβ.

### TRα and TRβ are of Opposing Prognostic Significance in BRCA1 Related Breast Cancer

**Fig 4:**
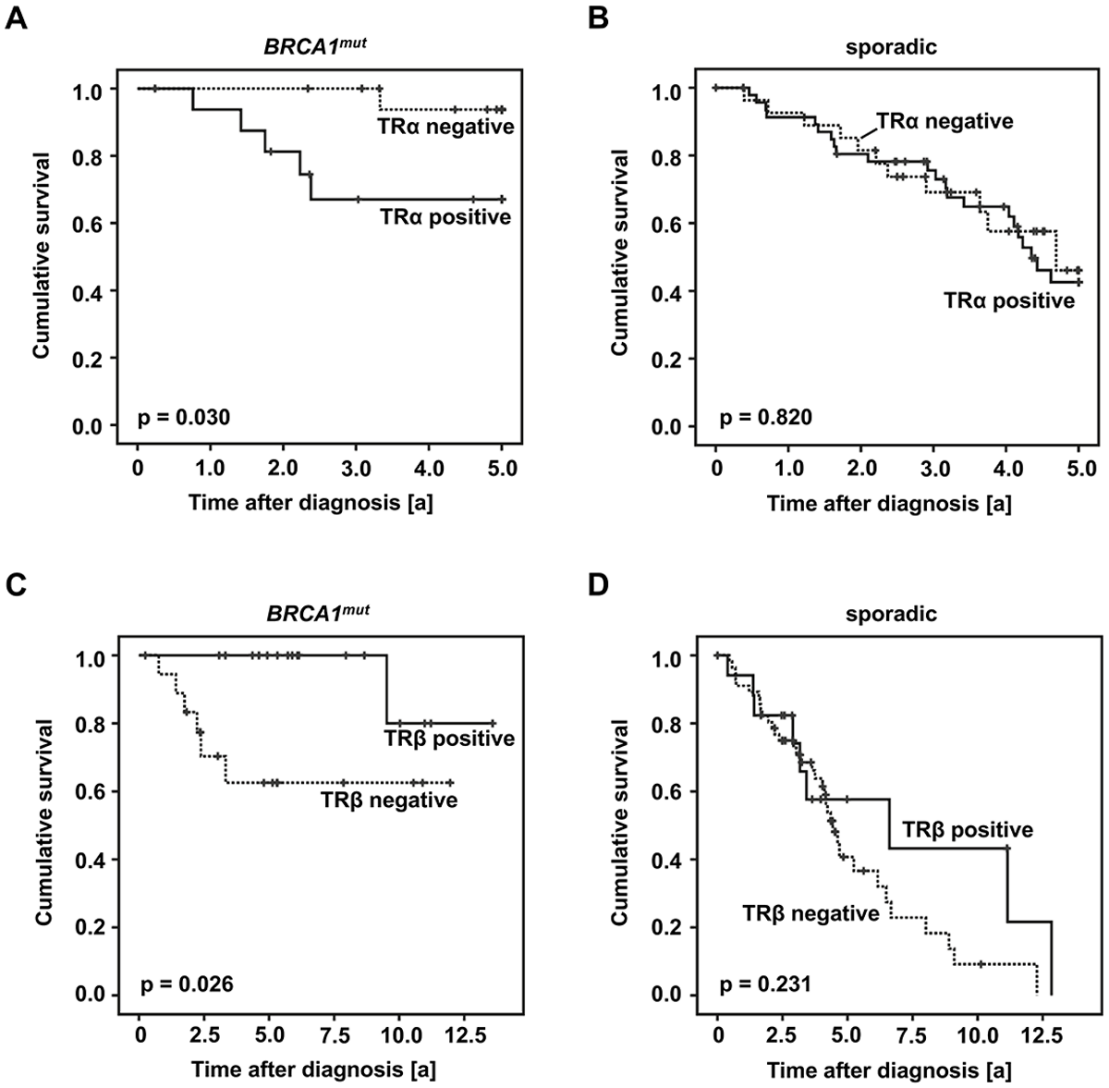
TR immuno-positivity predicts prognosis. Survival of TRα positive vs. negative cases (A, B) and TRβ positive vs. negative cases (C, D) was plotted in accordance with Kaplan-Meier survival curves. TRα was a negative predictor regarding five-year survival of patients carrying a *BRCA1* mutation (A). In sporadic cancer cases TRα was not associated with five-year survival (B). TRβ positivity was associated with a significantly prolonged overall survival in patients carrying a *BRCA1* mutation (C). In sporadic cancer cases TRβ failed to predict prognosis (D).

Five year survival of TR positive vs. negative cases was compared. TRα positivity was associated with significantly reduced five-year survival in *BRCA1* carriers (p = 0.030) (Fig 4A), while no effect of TRα on patient survival was observed in sporadic cancer cases (Fig 4B). In contrast, *BRCA1* associated cancers characterized as TRβ positive presented a significantly higher five-year survival rate as compared to TRβ-negative patients (p = 0.007), while TRβ failed to be of prognostic significance in sporadic breast cancer. Regarding *BRCA1* associated cancer cases neither TRα nor TRβ presented a significant association to any of the clinicopathological variables examined (Table 1). However, to reduce the effect of possible confounders, survival analysis was also performed in selected subgroups classified as NST, high grade, pN1-3, presence of metastasis, ER negative, PR negative and patient age below 42 years. TRα and TRβ remained to be predictive for five-year survival (TRα positivity—reduced five-year survival; TRβ positivity—advanced five-year survival) in breast cancer of non-specific type (TRα: p = 0.024; TRβ: p = 0.014), in high grade cancer (TRα: p = 0.042; TRβ: p = 0.005) and in patients aged younger than 42 years (TRα: p = 0.045; TRβ: p = 0.019). Further, TR positivity was also associated with five-year survival (TRα positivity—reduced five-year survival; TRβ positivity—advanced five-year survival) in ER negative (TRα: p = 0.032; TRβ: p = 0.020), PR negative (TRα: p = 0.011; TRβ: p = 0.029) *BRCA1* mutated breast cancer. Finally, TRβ positivity predicted a higher 5-year survival rate in metastasized (TRβ: p = 0.036) or lymph node positive (TRβ: p = 0.031) *BRCA1* mutated cancer.

More importantly, TRβ positivity predicted longer overall survival in *BRCA1* mutated patients (p = 0.026) (Fig 4C). Again, no relation was found between TRβ and overall survival in sporadic breast cancer (Fig 4D). TRβ remained to be a positive prognostic factor in *BRCA1* mutated cancers classified as NST (p = 0.036), high grade (p = 0.018), ER negative (p = 0.020) or PR negative (p = 0.029). However, though TRβ was also related to favorable prognosis in metastasized cancers (p = 0.036), it failed to be of prognostic significance in lymph node positive (p = 0.108) cases. The association of TRα positivity and reduced overall survival rate of *BRCA1* related cases was of borderline significance (p = 0.064).

Regarding the matched group, TRβ remained to be predictive for advanced overall survival in *BRCA1* associated cases (p = 0.018), while survival rates of TRβ positive vs. negative cases did not differ among sporadic breast cancers. A trend of TRα positivity being associated with reduced overall survival (p = 0.059) was observed in patients of the matched group carrying a *BRCA1* germline mutation. Again TRα was not predictive for sporadic breast cancer overall survival.

### TRs are active in *BRCA1* mutant HCC3153

**Fig 5:**
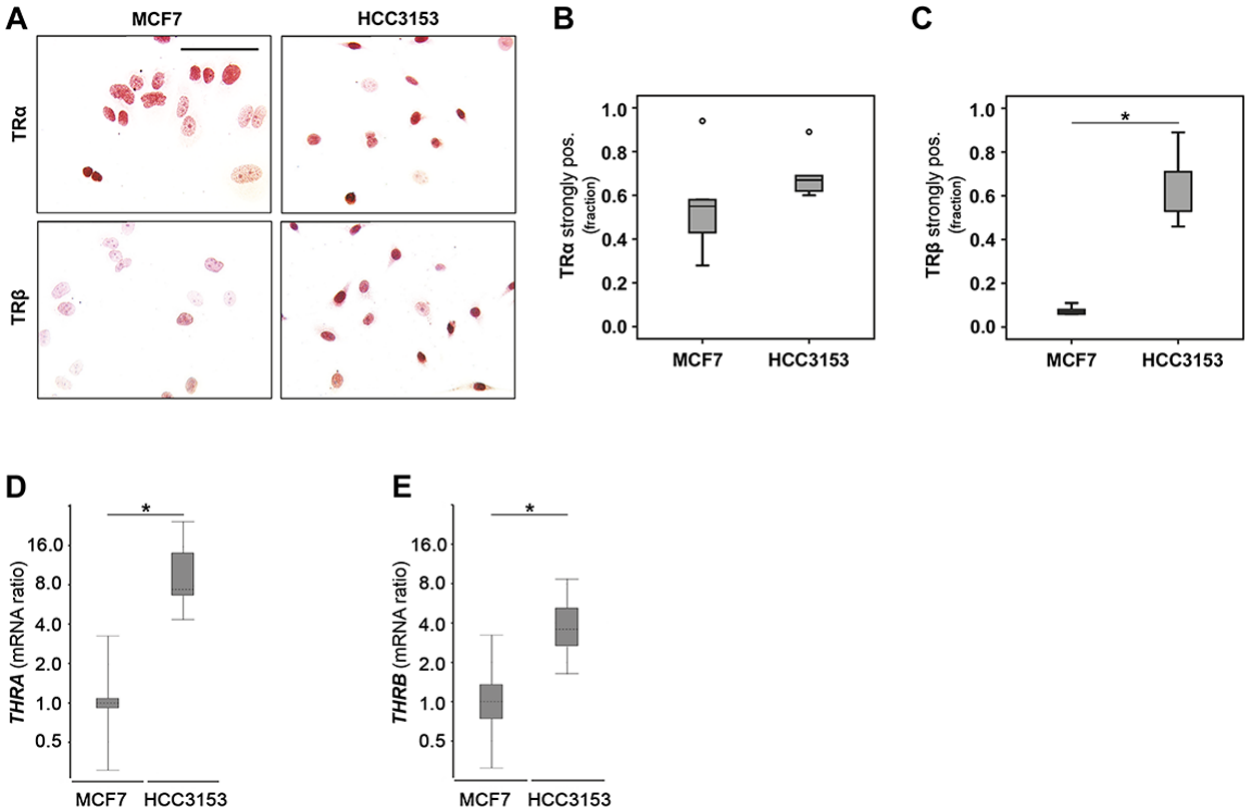
TRβ is strongly expressed in the absence of functional *BRCA1*. TRβ was significantly over-expressed on protein (A, C) and mRNA level (E) when BRCA1 competent MCF7 and HCC3153 carrying stop mutation in *BRCA1* were compared. However, in the case of TRα this difference was significant on mRNA level only (A, B, D). Mann-Whitney U Tests (B, C) and the REST2009 algorithm for determining relative gene expression (D, E) have been applied. Significant changes are indicated by stars (*) and representative images are shown.

TRβ overexpression in BRCA1 mutant cases was confirmed on protein and mRNA level in HCC3153 carrying a homozygous insertion in *BRCA1* as compared to *BRCA1* competent MCF7 (Fig 5). In line with our observation made in sporadic vs. *BRCA1* associated patients TRβ protein positivity rate was increased 8.4-fold in HCC3153 as compared to MCF7 (p = 0.009) (Fig 5A and 5C). *THRB* mRNA was increased in HCC3153 by a mean factor of 3.7 (S.E. range: 2.3–7.6) as compared to MCF7 (p = 0.034) (Fig 5E). Though there was no difference regarding TRα immunopositivity (Fig 5A and 5B), *THRA* mRNA was found to be elevated in HCC3153 by a mean factor of 9.3 (S.E. range: 5.3–20, p < 0.001) (Fig 5D).

**Fig 6:**
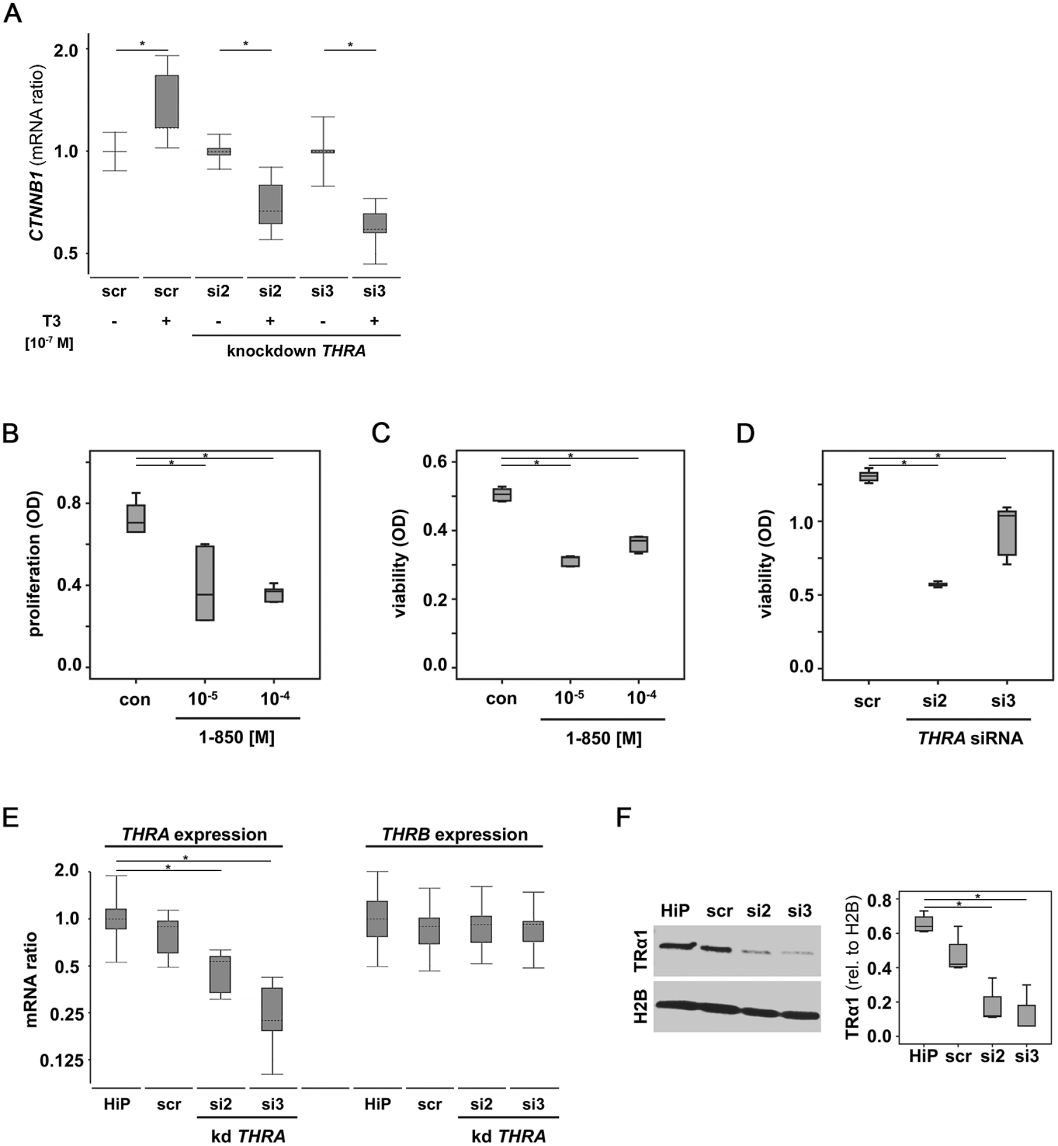
TRs are active in the absence of functional *BRCA1*. The TR target gene *CTNNB1* encoding β-Catenin was quantified in HCC3153 (*BRCA1* ^mut^) upon T3 stimulation. While T3 induced *CTNNB1* in HCC3153 expressing *THRA* as well as *THRB*, *CTNNB1* expression was significantly down-regulated upon T3 stimulation in HCC3153 silenced for *THRA* (A). Blocking TRs significantly reduced proliferation (B) and viability (C) of HCC3153 as quantified by BrdU (B) and WST-1 (C) assay, respectively. Reduced viability was also observed in HCC3153 silenced for *THRA* (D). Efficiency of *THRA* knockdown was validated on mRNA (E) and protein (F) level. *THRB* mRNA was not significantly altered in cells silenced for *THRA* (E). Mann-Whitney U Tests (B-D, F) and the REST2009 algorithm for determining relative gene expression (A, E) have been applied. Significant changes are indicated by stars (*) representative images are shown. scr—scrambled siRNA control, HiP—HighPerFect transfection reagent only control.

We further questioned whether TRs are active in the *BRCA1* deficient HCC3153 cell line. TRβ activation was shown to repress *CTNNB1*, the gene encoding tumor promoting β-catenin [22], while TRα activation was reported to induce *CTNNB1* [23]. Hence T3 stimulation was performed in HCC3153 silenced for *THRA* as well as in HCC3153 transfected with an off-target control siRNA. In scrambled control (scr) transfected cells treated with T3 *CTNNB1* expression was induced by a mean factor of 1.3 (S.E. range: 1.1–1.9, p < 0.001) while those HCC3153 silenced for *THRA* appeared to repress *CTNNB1* when stimulated with T3. Both *THRA* specific siRNAs were able to reverse the T3 effect on *CTNNB1* observed in scr treated cells resulting in repression of *CTNNB1* by a mean factor of si2: 0.69 (S.E. range: 0.61–0.86; p = 0.023) or si3: 0.60 (S.E. range: 0.54–0.70; p = 0.034) in those cells silenced for *THRA* and at the same time treated with T3 (Fig 6A).

TRα has been reported to act as a proliferation factor [24]. When HCC3153 were treated with the non-selective TR blocker 1–850 their proliferation (as quantified by BrdU incorporation) and viability (as quantified by WST-1 turnover) was found to be significantly reduced as compared to carrier solution (DMSO) treated samples. Median reduction of proliferation was 0.50 (p = 0.004) in samples treated with 10^-5^ M of 1–850 and 0.52 (p = 0.004) when cells were incubated with 1–850 at a concentration of 10^-4^ M (Fig 6B). Median viability was also decreased in HCC3153 stimulated with 1–850 (10^-5^ M: 0.64-fold, p = 0.004; 10^-4^ M: 0.73-fold, p = 0.004) (Fig 6C). Knocking down *THRA* in HCC3153 significantly lowered median cell viability (si2: 0.43-fold, p = 0.004; si3: 0.79-fold, p = 0.004) as determined by WST-1 assay (Fig 6D).

Knockdown efficiency was validated and *THRA* mRNA was significantly reduced in samples silenced for *THRA* (si2: 0.47-fold (S.E. range: 0.33–0.61), p = 0.027; si3: 0.24-fold (S.E. range is 0.17–0.39), p = 0.024) as compared to transfection reagent treated samples (HiP) (Fig 6E). *THRA* mRNA was also significantly repressed in *THRA* silenced samples (si2: 0.59-fold (S.E. range: 0.55–0.66), p = 0.033; si3: 0.31-fold (S.E. range is 0.19–0.43), p < 0.001) when compared to samples that had been transfected with the off target control (scr) (Fig 6E). *THRA* knockdown did not affect *THRB* mRNA expression (Fig 6E). Samples silenced for *THRA* showed significantly reduced TRα protein (Fig 6F) as compared to HiP treated (median reduction (si2): 0.27-fold, p = 0.02; median reduction (si3): 0.18-fold, p = 0.02) and as compared to scr transfected (median reduction (si2): 0.36-fold, p = 0.02; median reduction (si3): 0.24-fold, p = 0.02) samples.

### *BRCA1* mutant cells fail to degrade TRα1

**Fig 7:**
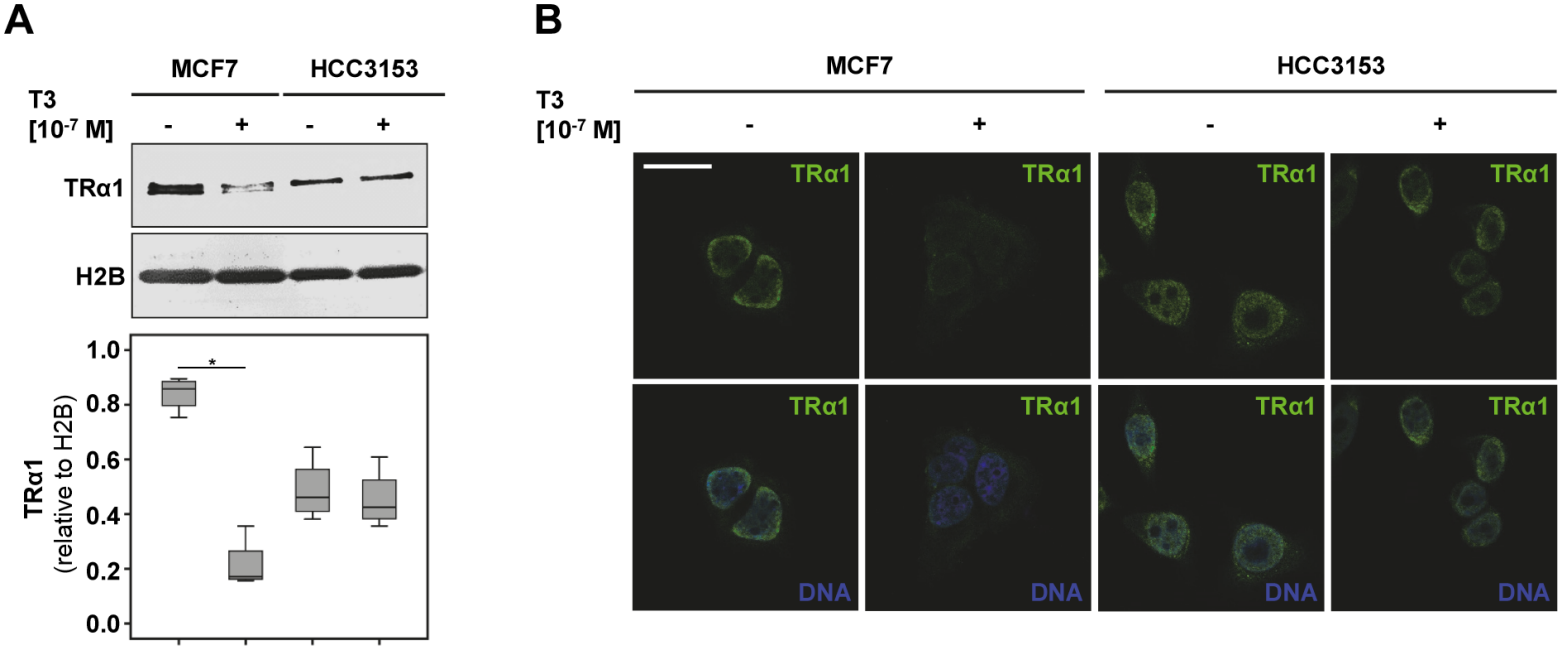
TRα1 positivity is reduced in MCF7 upon T3 stimulation. While BRCA1 competent MCF7 cells significantly lost TRα1 positivity in response to T3 stimulation, no such phenomenon was obvious in HCC3153 (A). Double immunofluorescence was performed (TRα1: green signal, DNA: blue signal). When stimulated with T3 subcellular localisation of TRα1 changed from a former predominant nuclear signal to a diffuse nuclear-cytoplasmic staining pattern (B). No such change in TRα1 localisation was visible in HCC3153 (B). Scale bar represents 25 μm. Significant changes as determined by relevant Mann-Whitney U Tests are indicated by stars (*) and representative images are shown.

Wildtype *BRCA1* has been reported to regulate protein half-life of nuclear hormone receptors e.g. progesterone receptor via its ubiquitinilation and sumoylation activity [25]. Though TRs are regulated by ubiquitinilation as well, it is not known whether this effect is reliant on functional BRCA1. Though *BRCA1* competent MCF7 cells significantly lost TRα1 positivity (median reduction: 0.20-fold, p = 0.021) in response to T3 stimulation, no such phenomenon was obvious in HCC3153 (Fig 7A). When stimulated with T3, subcellular localisation of TRα1 changed from a former predominant nuclear signal to a diffuse nuclear-cytoplasmic staining pattern. No such change in TRα1 localisation was visible in HCC3153 (Fig 7B).

**Fig 8:**
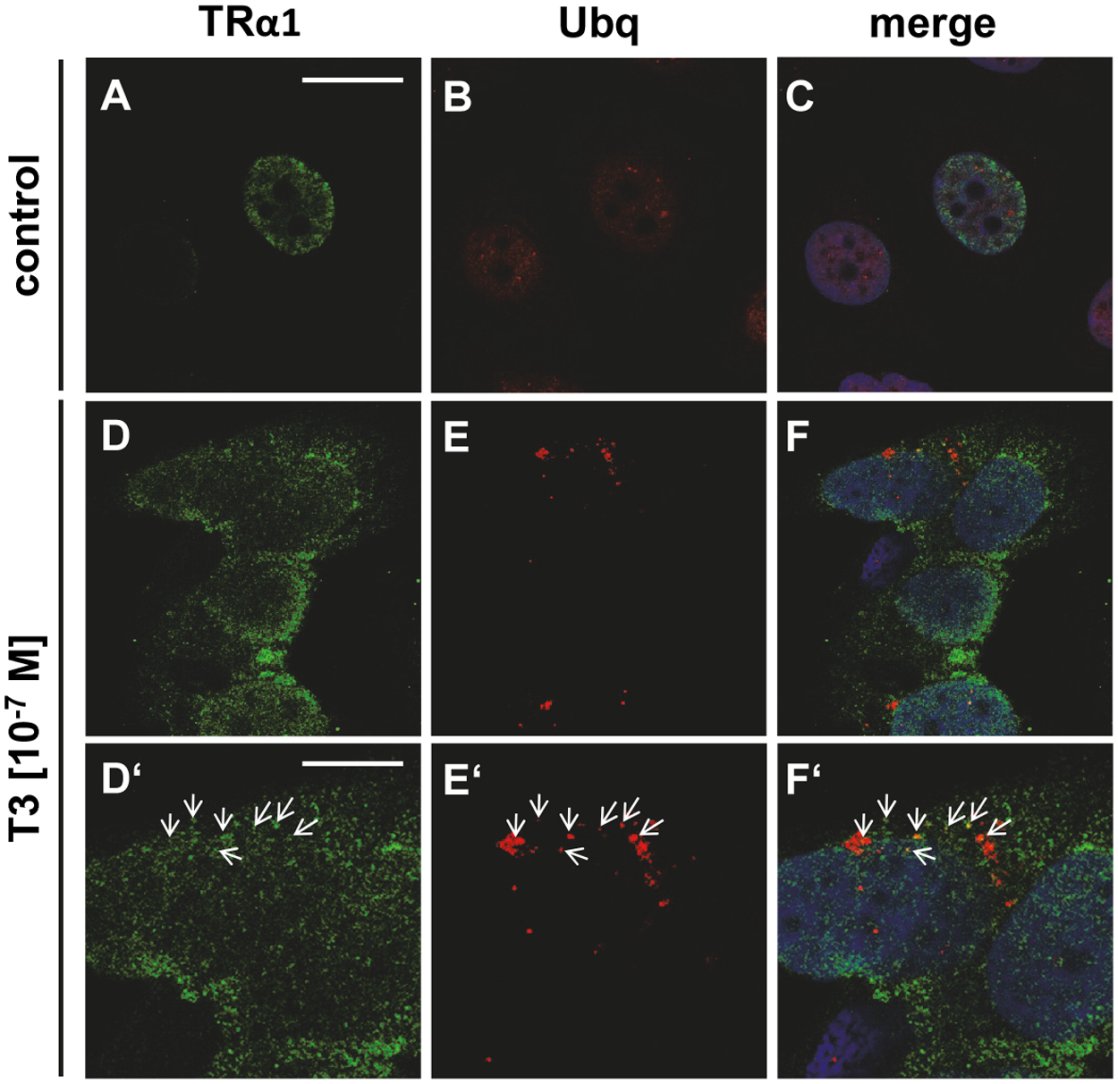
TRα1 co-localizes with ubiquitin upon T3 stimulation. BRCA1 competent MCF7 were co-stained for TRα1 (green signal) and ubiquitin (Ubq, red signal). TRα1 was located in the nucleus (DNA: blue signal) of untreated MCF7 (A, C). The red channel was slightly enhanced in B, C thus to show the faint Ubq signal of un-stimulated MCF7 (B, C). In BRCA1 competent MCF7 co-localization of TRα1 and Ubq was observed in samples stimulated with T3 (D-F, magnified in D’-F’). Scale bar in A equals 25 μm applies to A-F, scale bar in D’ equals 12.5 μm applies to D’-F’. Representative images are shown.

Upon T3 treatment some co-localisation of TRα1 and ubiquitin was observed in *BRCA1* competent MCF7 (Fig 8A–8F, magnification in D’-F’), potentially suggesting an ubiquitinilation mediated degradation process.

## Discussion

This work showed TRβ to be more frequently expressed in *BRCA1* associated breast cancers as compared to sporadic breast cancer. TRα and TRβ were observed to be of opposing prognostic significance regarding five year survival in *BRCA1* mutation carriers. In addition, TRβ positivity remained to be significantly associated with overall survival. Using cell lines we were able to show that TRs are active in a *BRCA1* mutant genetic background.

While a direct link is still missing, thyroid hormones and breast cancer have been associated for quite a while; e. g. an elevated incidence of thyroid dysfunction has been observed in breast cancer patients [26–28]. While several studies frequently found relative hyperthyreosis in breast cancer patients [26,29–31], others report aggravating hypothyroidism during breast cancer therapy or demonstrated an increased prevalence of autoimmune thyroid disorders in breast cancer [27,32]. In line with this, a former prospective clinical trial of our group revealed higher levels of auto-immune antibodies targeting thyroid hormone receptors (TRAKs) and of thyroidal effector hormones (triiodothyronine (T3), Thyroxine (T4)) in breast cancer patients as compared to controls [26]. Thyroid hormones did not correlate with TR expression in the current analysis. However, to the best of our knowledge no data exist upon thyroid function in *BRCA1* associated breast cancers so far. At least in the case of estrogen and ERs, nuclear hormone receptor expression does not seem to be related to circulating hormone levels [33]. More important, though anti-estrogen endocrine treatments are widely applied to ER positive patients, circulating estrogens are not accessed during clinical routine indicating that even if circulating estrogens alter ER expression this does not seem to be of clinical relevance.

Our current analysis on TRs indicated opposing roles of TRα and TRβ on survival of women carrying a *BRCA1* germline mutation and on target gene activation in *BRCA1* mutant cancer cells. Interestingly, a couple of other *in vitro* cell culture studies also report a tumor-promoting effect of TRα activation [34–37], while TRβ stimulation resulted in just the opposite [38–40]. For instance, quite recently TRα was found to enhance cancer cell migration and metastasis via inhibition of miR-17 [34] as well as to maintain pro-neoplastic potency by interacting with the cyclin D1/CDK/Rb/E2F cascade [35], the wnt pathway [3] or by inducing *CTNNB1* gene expression [23]. Since our data demonstrate that selective THRA knockdown inhibited cell growth, a tumor promoting action of TRα is further affirmed. In contrast, TRβ exerted anti-tumor activity by inhibiting beta catenin action [39] or by interfering with AKT-mTOR-p70S6K signaling in xenografted mice [38]. In line with our data on differential regulation of *CTNNB1* in *BRCA1* deficient HCC3153 either expressing both TRα and TRβ or just expressing TRβ (i.e. silenced for *THRA*) further supports an opposing role of TRs in breast cancer [22]. Simultaneous blockade of TRα and TRβ by the non-selective TR antagonist 1–850 reduced cell growth potentially indicating that inhibition of pro-proliferative TRα may outweigh TRβ mediated effects. Interestingly, regarding both *CTNNB1* expression and cell growth TRα effects seem to dominate the influence of TRβ.

Surprisingly, TRs demonstrated prognostic significance only in those patients diagnosed for a *BRCA1* germline mutation but not in sporadic breast cancer cases. This is in agreement with reports where neither TRα nor TRβ were significantly associated with patient prognosis in a different, independent sample of 82 sporadic breast cancers [6]. Although experimental evidence explaining the impact of TRs in *BRCA1* associated cancers is still lacking, the current study showed TRβ to be more frequently expressed in *BRCA1* mutation carriers. This was confirmed by comparing TRβ expression on both mRNA and protein level in *BRCA1* mutant HCC3153 vs. *BRCA1* competent MCF7. Immuno-positivity of TRα was not significantly different when *BRCA1* mutant and sporadic breast cancers were compared though *THRA* mRNA expression was induced in HCC3153. This may be explained by the fact that correlation of protein and mRNA expression of certain genes has been reported to be rather poor [41–45]. The latter phenomenon may be due to complex post-transcriptional regulatory processes, many of which have not been sufficiently understood so far. In addition the probability of a direct correlation between mRNA and protein seems to be dependent on e.g. ribosomal occupancy, protein stability, codon usage or on whether mRNA concentrations of a certain ORFs [42,43]. Since the wildtype BRCA1 protein contributes to protein degradation via its ubiquitinilation and sumoylation activity of nuclear hormone receptors [25], loss of functional *BRCA1* may explain TRβ overexpression and prognostic relevance of both TRs. This hypothesis is supported by increasing evidence of ubiquitinylation and sumoylation mediated regulation of TRs upon hormone binding [46,47]. Our results on degradation of TRα1 may indicate that BRCA1 is involved in posttranscriptional regulation of TRs. However co-immunoprecipitation assays will be needed to confirm a physical interaction of TRα1 and Ubiquitin. *BRCA1* mutants may be used to confirm whether reduced TRα1 degradation observed in HCC3153 may indeed be reliant on *BRCA1*. Since, from a clinical point of view, activation of a tumor suppressor gene is less attractive than blocking an oncogene, only posttranscriptional regulation of TRα1 was studied.

Survival analysis, cell growth and target gene activation assays performed within this study indicate that TRs not only hold prognostic significance but also are active in a *BRCA1* deficient genetic background. Therefore we hypothesize that *BRCA1* mutant cells may undergo a TR mediated phenomenon termed ‘oncogenic addiction’ [48]. Within this scenario, cancer cells acquire abnormalities in several onco (e.g. TRα)—and tumor suppressor (e.g. TRβ) genes. Gene products being crucial for cancer cell survival provide an Achilles heel for tumors and display interesting targets to be exploited in cancer treatment [48]. In case of *BRCA1* associated breast cancers, TRs might display excellent targets for novel drugs, especially in triple-negative cases. In line with this, the current study demonstrated TRs to be expressed in 9 out of 12 triple negative cancers and to be highly sensitive to TR modulation in triple negative, *BRCA1* mutant HCC3153.

Within the last decade a couple of selective as well as non-selective TR modifiers have been investigated. Dronedarone was found to inhibit TRα *in vitro* as well as *in vivo* [49] and a study in *Xenopus laevis* reported the TRα stimulating compound CO23 to support brain cell proliferation, while such induction of proliferation was not seen in animals treated with the TRβ selective agonists GC1 or GC24 [24].

In conclusion, our work revealed at least that TRs are active in *BRCA1* associated breast cancer, that TRβ expression in *BRCA1* mutant tumor samples is associated with a prolonged overall survival, and that both TRs may arise as interesting alternative targets for endocrine treatment of *BRCA1* associated triple negative breast cancer.
